# Genotype–Phenotype Correlations in Children with HHT

**DOI:** 10.3390/jcm9092714

**Published:** 2020-08-22

**Authors:** Alexandra Kilian, Giuseppe A. Latino, Andrew J. White, Dewi Clark, Murali M. Chakinala, Felix Ratjen, Jamie McDonald, Kevin J. Whitehead, James R. Gossage, Doris Lin, Katharine Henderson, Jeffrey Pollak, Justin P. McWilliams, Helen Kim, Michael T. Lawton, Marie E. Faughnan, the Brain Vascular Malformation Consortium HHT Investigator Group

**Affiliations:** 1 Toronto HHT Centre, St. Michael’s Hospital and Li Ka Shing Knowledge Institute, Toronto, ON M5B 1W8, Canada; alexandra.kilian@mail.utoronto.ca (A.K.); Giuseppe.Latino@nygh.on.ca (G.A.L.); dewi.clark@unityhealth.to (D.C.); 2 Department of Pediatrics, North York General Hospital, University of Toronto, Toronto, ON M2K 1E1, Canada; 3 Department of Pediatrics, Washington University School of Medicine, St Louis, MO 63110, USA; white_a@wustl.edu; 4 Division of Respirology, Department of Medicine, University of Toronto, Toronto, ON M5S 3H2, Canada; 5 Division of Pulmonary and Critical Care Medicine, Washington University School of Medicine, St Louis, MO 63110, USA; chakinalam@wustl.edu; 6 Division of Respiratory Medicine and Translational Medicine, The Hospital for Sick Children, Toronto, ON M5G 1X8, Canada; felix.ratjen@sickkids.ca; 7 Department of Pathology, University of Utah, Salt Lake City, UT 84132, USA; jamie.mcdonald@hsc.utah.edu; 8 Department of Medicine, Division of Cardiovascular Medicine, University of Utah, Salt Lake City, UT 84132, USA; kevin.whitehead@hsc.utah.edu; 9 Department of Pediatrics, Division of Pediatric Cardiology, University of Utah, Salt Lake City, UT 84132, USA; 10 Department of Medicine, Augusta University, Augusta, GA 30912, USA; jgossage@augusta.edu; 11 Department of Radiology, The Johns Hopkins University School of Medicine, Baltimore, MD 21205, USA; ddmlin@jhmi.edu; 12 Department of Radiology and Biomedical Imaging, and HHT Program, Yale University School of Medicine, New Haven, CT 06510, USA; katharine.henderson@yale.edu (K.H.); jeffrey.pollak@yale.edu (J.P.); 13 Department of Interventional Radiology, University of California Los Angeles, Los Angeles, CA 90095, USA; jumcwilliams@mednet.ucla.edu; 14 USA Center for Cerebrovascular Research, Department of Anesthesia and Perioperative Care, University of California San Francisco, San Francisco, CA 94110, USA; Helen.Kim2@ucsf.edu; 15 Institute for Human Genetics, University of California San Francisco, San Francisco, CA 94143, USA; 16 Department of Epidemiology and Biostatistics, University of California San Francisco, San Francisco, CA 94158, USA; 17 Department of Neurosurgery, Barrow Neurological Institute, Phoenix, AZ 85013, USA; michael.lawton@barrowbrainandspine.com

**Keywords:** Hereditary hemorrhagic telangiectasia, pediatrics, genotype–phenotype correlation, arteriovenous malformation, *ENG*, *ACVRL1*, *SMAD4*

## Abstract

Hereditary hemorrhagic telangiectasia (HHT), a rare autosomal dominant disease mostly caused by mutations in three known genes (*ENG*, *ACVRL1*, and *SMAD4*), is characterized by the development of vascular malformations (VMs). Patients with HHT may present with mucocutaneous telangiectasia, as well as organ arteriovenous malformations (AVMs) of the central nervous system, lungs, and liver. Genotype–phenotype correlations have been well described in adults with HHT. We aimed to investigate genotype–phenotype correlations among pediatric HHT patients. Demographic, clinical, and genetic data were collected and analyzed in 205 children enrolled in the multicenter Brain Vascular Malformation Consortium HHT Project. A chi-square test was used to determine the association between phenotypic presentations and genotype. Among 205 patients (age range: 0–18 years; mean: 11 years), *ENG* mutation was associated with the presence of pulmonary AVMs (*p* < 0.001) and brain VM (*p* < 0.001). The presence of a combined phenotype—defined as both pulmonary AVMs and brain VMs—was also associated with *ENG* mutation. Gastrointestinal bleeding was rare (4.4%), but was associated with *SMAD4* genotype (*p* < 0.001). We conclude that genotype–phenotype correlations among pediatric HHT patients are similar to those described among adults. Specifically, pediatric patients with *ENG* mutation have a greater prevalence of pulmonary AVMs, brain VMs, and a combined phenotype.

## 1. Introduction

Hereditary hemorrhagic telangiectasia (HHT) is a rare autosomal dominant disease affecting approximately 1 in 5000 people [1,2,3,4]. HHT can be diagnosed clinically using the Curaçao clinical diagnostic criteria [5] or by genetic testing [6]. Mutations in the endoglin (*ENG*) and activin A receptor-like kinase 1 (*ACVRL1*) genes account for approximately 96% of cases, when the Curaçao clinical diagnostic criteria are strictly applied [7]. In addition, less than 2% of patients present with an HHT–Juvenile Polyposis (JP) overlap syndrome, caused by mutations in the *SMAD4* gene [7,8,9]. HHT is characterized by the development of arteriovenous malformations (AVMs) in visceral organs, including the brain, lungs, liver, and rarely the spine. AVMs carry risks of life-threatening complications, including hemorrhage and paradoxical embolisms [6,10,11]. Additionally, smaller vascular malformations—called telangiectases—occur on mucocutaneous surfaces [11]. Bleeding from telangiectases in the nasal mucosa results in spontaneous recurrent epistaxis. Additionally, patients can have chronic bleeding from telangiectases in the gastrointestinal (GI) mucosa, often complicated by secondary iron-deficiency anemia [12].

Genotype–phenotype correlations have been well described in adult cohorts [10,13,14,15,16,17,18,19]. Most conclusively, the *ENG* genotype (HHT1: OMIM# 18730) has been associated with the presence of pulmonary AVMs [10,13,14,15,17,19,20,21] and brain VMs [10,13,14,15,16,21] in adults. While there are multiple brain VM subtypes seen in HHT, including arteriovenous fistulas (AVFs), nidus-type AVMs, and capillary vascular malformations, no correlations between brain VM subtype and genotype have been described [22]. The *ACVRL1* mutation (HHT2: OMIM# 600376) has been associated with the presence of liver VMs [10,13,14,20,21]. However, there is a relative paucity of literature regarding genotype–phenotype correlations, as well as HHT manifestations and complications in pediatric patients. Smaller pediatric cohorts have demonstrated that the *ENG* genotype is correlated with pulmonary AVMs and brain VMs [23,24,25]. The visible features of HHT—mucocutaneous telangiectases and spontaneous recurrent epistaxis—increase with age [26,27,28]. This, combined with the rarity of the disease, can result in delayed presentation and diagnosis in children [5,6,29,30]. Thus, a more robust understanding of genotype–phenotype correlations will have implications for diagnosis of HHT and organ screening in children. Accordingly, we aimed to report data from a large pediatric cohort of patients with HHT and describe the genotype-phenotype correlations.

## 2. Materials and Methods

### 2.1. Cohort

Demographic, clinical, and genetic data were collected from 205 pediatric patients (age at recruitment ≤18) enrolled in the Brain Vascular Malformation Consortium (BVMC) HHT Project. The BVMC HHT Project includes 1679 HHT patients with a definite clinical or genetic diagnosis of HHT, enrolled at multiple recruiting centers in the US, Canada, and the Netherlands between 2010 and 2019. Cohort recruitment has been previously described [18,31]. The 205 pediatric patients in this study were recruited from nine of the BVMC recruiting centers. Informed written consent was obtained from all patients to be included in all BVMC related projects. The study protocol was approved by the institutional review board at each recruiting center (SMH REB#09-212 for lead site at St. Michael’s Hospital). Patients were screened for organ VMs and other clinical features according to standard clinical practice (not as study procedure) and International HHT Guidelines [6]. Organ VM screening typically included: comprehensive history, physical exam, and routine investigations; pulmonary AVMs screening with contrast echocardiography or positional oximetry (protocols varied by center); brain VM screening by magnetic resonance imaging (MRI); clinical screening for liver VMs (chronic right upper quadrant pain, portal hypertension, high-output heart failure, liver bruit on examination); clinical screening for recurrent spontaneous epistaxis (>1 episode per month for >1 year); and screening for HHT-related GI bleeding (history of anemia, iron deficiency, known GI telangiectases on endoscopy, melena, rectal bleeding). If screening was positive for pulmonary AVM or brain VM, patients underwent confirmatory diagnostic imaging and treatment where appropriate. If clinical assessment was suggestive of symptomatic liver VM, diagnostic imaging was recommended and therapy was initiated where appropriate. Finally, if the initial clinical assessment was suggestive of GI bleeding, diagnostic endoscopy was recommended and endoscopic, medical, and supportive therapies were undertaken on a case-by-case basis. The BVMC HHT cohort targets 25% of brain VM-positive patients, while other characteristics are similar to other cohorts [13,16].

### 2.2. Analysis

We tested whether HHT genotype (*ACVRL1*, *ENG*, *SMAD4*) was associated with clinical features including the presence of epistaxis, typical mucocutaneous telangiectasia, anemia, pulmonary AVMs, brain VMs, GI bleeding, and symptomatic liver VMs. For the purposes of our analysis, patients with micro-pulmonary AVMs were considered to be negative for pulmonary AVMs. All brain VM subtypes met the criteria for the brain VM phenotype. A combined phenotype was defined as the presence of both pulmonary AVM(s) and brain VM(s). Statistical analysis was conducted using the SPSS version 21.0.0. All *p*-values calculated were two-sided and significance was defined at *p* < 0.05. The Kruskal–Wallis test was used to compare the association between continuous variables (age) and genotype. To determine the association between clinical features and genotype (*ACVRL1*, *ENG*, *SMAD4*), a chi-square test was used.

## 3. Results

Table 1 shows demographic and clinical characteristics of the 205 pediatric HHT patients comprising our cohort, as well as the phenotype characteristics by genotype. Our cohort was 47% female with an average age of 9.9 years. There was no significant difference in the distribution of age or sex between the three genotypes. Among the 205 patients, 176 (85.9%) had epistaxis, 107 (52.2%) had typical mucocutaneous telangiectases, 62 (30.2%) had at least one pulmonary AVM, seven (3.4%) had symptomatic liver VMs, and 70 (34.1%) had one or more brain VM. There was evidence of GI bleeding in nine patients (4.4%) and 30 patients (14.6%) were anemic.

### 3.1. Genotype

A total of 171 (83.4%) patients in our cohort had a known genetic mutation, confirmed by genetic testing of the patient (156/171 (91.2%)) or a family member (15/171 (8.8%)). The *ENG* genotype was the most common in our cohort, present in 101 of 171 patients (59.1%) with a known mutation. In our cohort, 6/162 (3.7%) patients who underwent genetic testing did not have an identifiable mutation.

### 3.2. Epistaxis

The most common clinical manifestation of HHT in pediatric patients was spontaneous recurrent epistaxis, present among 172/205 (83.9%) of our patients. There was no significant association between genotype and the presence of epistaxis (*p* = 0.865). 

### 3.3. Mucocutaneous Telangiectases

Approximately half (50.7%) of patients in our cohort had typical mucocutaneous telangiectases. The prevalence by genotype was almost equivalent between patients with *ENG* and *ACVRL1* mutations at 46.5% and 47.5%, respectively. Four (36.4%) patients with a *SMAD4* mutation had typical mucocutaneous telangiectases. There was no significant association between genotype and the presence of telangiectasia (*p* = 0.790).

### 3.4. GI Bleeding

A history of GI bleeding was reported in 9/205 (4.4%) patients. Patients with a *SMAD4* mutation were significantly more likely to experience GI bleeding (*p* < 0.001), compared to patients with *ENG* or *ACVRL1* mutations. Clinical and endoscopic data was available in 7/9 (77.8%) patients with GI bleeding. All 7/7 (100%) had lower GI bleeding; none reported upper GI bleeding. In 5/7 (71.4%) patients, polyps were identified as the cause of GI bleeding. No cause was identified in the remaining 2/7 (28.6%) patients with GI bleeding and GI bleeding resolved spontaneously

### 3.5. Anemia

A history of anemia was reported in 28/205 (13.7%) patients. No genotype was significantly associated with current or historical anemia (*p* = 0.735). Notably, history of anemia was not significantly associated with epistaxis or GI bleeding.

### 3.6. Organ Vascular Malformations

Pulmonary AVMs were reported in 62/205 (30.2%) patients in our cohort. Brain VMs were reported in 70/205 (34.1%) patients. Pediatric patients with an *ENG* mutation were significantly more likely to have pulmonary AVMs (*p* < 0.001) and brain VMs (*p* < 0.001). Patients with an *ENG* mutation were also more likely to have any organ VM (pulmonary, brain, liver) compared to patients with *ACVRL1* or *SMAD4* mutations (*p* < 0.001). While a higher proportion of patients with an *ACVRL1* mutation had liver VMs compared to patients with an *ENG* gene mutation, this was not statistically significant (*p* = 0.093).

### 3.7. Combined Phenotype

Thirty-three (16.1%) of patients in our cohort had a combined phenotype, characterized by the presence of both pulmonary AVM(s) and brain VM(s). The majority (72.7%) of the patients with this combined phenotype had an *ENG* mutation and this association was found to be statistically significant (*p <* 0.001). Notably, sex was not a risk factor for the combined phenotype; 17/33 patients (51.5%) with a combined phenotype were male.

## 4. Discussion

Here, we report data from a large pediatric cohort of patients with HHT and describe genotype–phenotype correlations. Most significantly, our data demonstrate that in pediatric patients, pulmonary AVMs and brain VMs are more frequent in patients with an *ENG* mutation, as reported in adult studies [10,13,14,15,17,19,20,21]. Moreover, the *ENG* genotype is associated with a combined phenotype, characterized by the presence of both pulmonary AVM(s) and brain VM(s), in pediatric patients. Finally, while GI bleeding is rare in pediatric HHT patients, it is associated with an *SMAD4* mutation and seen commonly in patients with this genotype.

In our cohort, *ENG* mutation was associated with increased prevalence of brain VMs and pulmonary AVMs. This is consistent with previous literature describing genotype–phenotype correlations in adults, in which the *ENG* genotype was associated with the presence of pulmonary AVMs [10,13,14,15,17,19,20,21] and with the presence of brain VMs [10,13,14,15,16,21]. Our results also confirm those of previous smaller pediatric series reported by Giordano et al. [23] and A-Saleh et al. [24], comprised of 44 and 61 patients, respectively. While certain clinical features of HHT are age-dependent, including telangiectasia and epistaxis, a similar prevalence of pulmonary AVMs in children as in adults has been reported [25]. This suggests that the pulmonary AVM phenotype is consistent across age groups and may not be age dependent [25].

These observations may have important implications for clinical care and diagnostics in HHT. First, given the similarities in genotype–phenotype correlations, children, like adults, are at risk of complications from organ VMs. Thus, organ screening should be considered. The rationale for pulmonary AVM screening is to identify children at risk of serious complications and who might benefit from preventative management with transcatheter embolization. The rationale for brain VM screening is similar; screening allows for the identification of children at risk of life-threatening and debilitating complications and allows for the consideration of preventative management. The decision to treat brain VMs is typically made on a case-by-case basis, with an expert neurovascular team, balancing the risks of the brain VM with those of the treatment. There is some controversy internationally regarding the role for brain VM screening, resulting in regional practice variation. In North America, the current standard of practice is to screen for brain VMs at the time of diagnosis using MRI. Currently, if initial screening for brain VMs is negative, in childhood, some centers repeat screening every five years or at least once in early adulthood [32].

Second, while children with an *ENG* mutation are at greater risk of having pulmonary AVMs, this feature is present across all genotypes, as in adults. There is, therefore, no evidence to support genotype based pulmonary screening recommendations. Similarly, while the literature suggests increased prevalence of brain VMs in adult patients with an *ENG* mutation [10,13,14,15,16,21], brain VMs have been described across all genotypes in adult patients [16]. Our observations are consistent with this; while an *ENG* mutation is associated with brain VM in children, brain VMs are also reported in children with an *ACVRL1* mutation. Although we reported no brain VMs in children with an *SMAD4* mutation, this may have been due to small numbers in this group. Notably, the presence of brain VMs in 34% of patients in our series is higher than previous adult and pediatric reports. Giordano et al. reported brain VM presence in 7/44 (15.9%) children [23]. In adult patients with HHT, the current data suggests that approximately 23% will have a brain VM [33,34,35]. The increased representation in our cohort was expected due to the explicit recruitment strategy of the BVMC HHT project, which targets recruiting HHT patients but with a 25% recruitment target for patients with brain VMs [31]. Third, as organ VMs are common in children, testing for their presence may be helpful in confirming the clinical diagnosis of HHT in children of families where genetics are not informative.

We also demonstrated that, overall, GI bleeding was a rare clinical manifestation in children with HHT, but was frequent in children with an *SMAD4* mutation. This is not surprising given the known overlap between HHT and Juvenile Polyposis (OMIM# 175050) in patients with *SMAD4* mutations [8,9]. The overall low prevalence of GI bleeding in our pediatric cohort is consistent with previous literature demonstrating later-life onset of GI bleeding in HHT patients [1,28,36]. In fact, Plauchu et al. reported that only 1.5% of patients had GI bleeding at ages younger than 30 [28]. In adults, anemia is a typical complication of chronic GI bleeding in HHT [6,36,37,38], and Kasthuri et al. described GI bleeding as an independent predictor of anemia in adult HHT patients [12]. Given the typically later-life onset of GI bleeding, the current HHT guidelines [6] recommend monitoring hemoglobin and hematocrit after the age of 35, as a marker of GI bleeding. Our data corroborates the appropriateness of delayed screening for GI bleeding in children with an *ENG* or *ACVRL1* mutation until adulthood. However, children with an *SMAD4* mutation should be considered at risk for earlier GI bleeding and secondary anemia.

Children with an *ENG* gene mutation are at higher risk of a combined phenotype, with the presence of both brain VM(s) and pulmonary AVM(s). The *ENG* genotype has been previously implicated in this combined phenotype in adults [13]. In our cohort, two patients with an *ACVRL1* mutation demonstrated a combined phenotype presentation. Additionally, a causative mutation was unknown in over 20% of cases. The frequency of the combined phenotype in our cohort (16.1%) is higher than the prevalence of 8.7% reported by Letteboer et al. in an adult population [13] and higher than the prevalence of 11.4% reported by Giordano et al. in a pediatric population [23]. Though our prevalence of the combined phenotype may have been overestimated due to selection bias, the association with an *ENG* mutation is highly significant and clinically important. In addition, the presence of the combined phenotype in non-HHT1 children was also an important observation, which once again suggests that organ VM screening in children with HHT should not be reserved for children with *ENG* mutations only. This is in line with the recommendations made by the International HHT guidelines, that genotype should not guide screening practices [6].

We believe our results can be generalized to children with HHT for several reasons. The clinical characteristics of the children in our cohort are similar to other previously published pediatric cohorts. In addition, the multi-center nature of the data supports generalizability. The *ENG* and *ACVRL1* genotype distribution in our data set—59% of patients with an *ENG* mutation, 35% of patients with an *ACVRL1* gene mutation—are in the usual range for North American and some European HHT populations [10,39,40]. In addition, our results align with genotype–phenotype correlations from earlier smaller pediatric cohorts [20,23,24,25], as well as the previously described trends in the adult population [10,13,14,15,16,21].

There are several limitations to our study that warrant discussion. Firstly, the prevalence of brain VMs in our cohort was intentionally enriched in the recruitment design. The BVMC HHT Project aims for 25% of patients with brain VMs. Thus, our cohort likely over represents the brain VM prevalence. Moreover, given that our cohort consisted of patients that were ≤18 years of age at the time of recruitment, we did not include patients diagnosed in childhood, but recruited in adulthood. Additionally, the data collection in the BVMC HHT project is retrospective. While we did not distinguish between brain VM subtypes, this is a topic of interest for future research. Finally, we collected and reported on the presence of anemia based on patient reports and chart review, but we did not collect laboratory data for confirmation or to classify type of anemia. Though this may have led us to underestimate the prevalence of anemia in the cohort, our observed low prevalence of anemia in children with HHT is in keeping with clinical experience [41]. Despite these limitations, the results remain robust and clinically important given the larger size of the patient cohort, the statistical significance of the genotype–phenotype correlations and the consistency of the results with adult observations in HHT.

## 5. Conclusions

From the largest genotype–phenotype cohort of pediatric patients with HHT to date, we demonstrate that organ involvement and associated genotype-phenotype correlations in children with HHT are similar to those previously described in the adult population. Specifically, the *ENG* genotype is associated with pulmonary AVMs and brain VMs in children with HHT. Moreover, pediatric patients can present with a combined phenotype, with both pulmonary AVM and brain VM, which is also associated with the *ENG* genotype. Our results highlight the importance of organ VM screening in pediatric patients with HHT.

## Figures and Tables

**Table 1:** Demographic and clinical characteristics of pediatric HHT patients.

Characteristic	All Patients *n* = 205	ENG (101/171, 59%)	ACVRL1 (59/171, 35%)	SMAD4 (11/171, 6%)	*p*-Value
Female Sex (%)	97 (47%)	51/101 (50.5%)	28/59 (47.5%)	2/11 (18.2%)	0.125
Mean Age (yrs) (±Standard Deviation (years))	9.9 (±6.5)	9.4 (±5.4)	9.2 (±5.5)	11.27 (±4.9)	0.430
Age range	1 month–18 years	1 month–18 years	1 month–18 years	4–17 years	–
Epistaxis	172/205 (83.9%)	86/101 (85.1%)	51/59 (86.4%)	10/11 (90.9%)	0.865
Telangiectasia	104/205(50.7%)	47/101 (46.5%)	28/59 (47.5%)	4/11 (36.4%)	0.790
Pulmonary AVM	62/205 (30.2%)	44/101 (43.6%)	4/59 (6.8%)	1/11 (9.1%)	<0.001
Brain VM	70/205 (34.1%)	45/101 (44.6%)	10/59 (17.0%)	0/11 (0%)	<0.001
GI bleeding	9/205 (4.4%)	2/101 (1.8%)	1/59 (1.7%)	5/11 (45.5%)	<0.001
Anemia	28/205 (13.7%)	13/101 (12.9%)	10/59 (17.0%)	2/11 (18.2%)	0.735
Liver VM	7/205 (3.4%)	1/101 (0.9%)	4/59 (6.8%)	0/11 (0%)	0.093
Any VM	103/205 (50.2%)	65/101 (64.4%)	17/59 (28.8%)	1/11 (0.9%)	<0.001
Combined phenotype ^1^	33/205 (16.1%)	24/101 (23.8%)	2/59 (3.4%)	0/10 (0%)	0.001

^1^ Combined phenotype: combined presence of both pulmonary AVM(s) and brain VM(s). HHT: hereditary hemorrhagic Telangiectasia; VM: vascular malformation; AVM: Arteriovenous malformation; GI: gastrointestinal.

## References

[1] Kjeldsen A.D., Vase P., Green A. (1999). Hereditary haemorrhagic telangiectasia: A population-based study of prevalence and mortality in Danish patients. J. Intern. Med..

[2] Donaldson J., McKeever T.M., Hall I.P., Hubbard R., Fogarty A.W. (2014). The UK prevalence of hereditary haemorrhagic telangiectasia and its association with sex, socioeconomic status and region of residence: A population-based study. Thorax.

[3] Sabba C., Pasculli G., Cirulli A., Gallitelli M., Virgilio G., Resta F., Guastamacchia E., Palasciano G. (2002). Hereditary hemorrhagic teleangiectasia (Rendu-Osler-Weber disease). Minerva Cardioangiol..

[4] Dakeishi M., Shioya T., Wada Y., Shindo T., Otaka K., Manabe M., Nozaki J.-I., Inoue S., Koizumi A. (2002). Genetic epidemiology of hereditary hemorrhagic telangiectasia in a local community in the northern part of Japan. Hum. Mutat..

[5] Shovlin C.L., Guttmacher A.E., Buscarini E., Faughnan M.E., Hyland R.H., Westermann C.J., Kjeldsen A.D., Plauchu H. (2000). Diagnostic criteria for hereditary hemorrhagic telangiectasia (Rendu-Osler-Weber syndrome). Am. J. Med. Genet. A.

[6] Faughnan M., Palda V., Garcia-Tsao G., Geisthoff U., McDonald J., Proctor D., Spears J., Brown D., Buscarini E., Chesnutt M. (2011). International guidelines for the diagnosis and management of hereditary haemorrhagic telangiectasia. J. Med. Genet..

[7] McDonald J., Bayrak-Toydemir P., DeMille D., Wooderchak-Donahue W., Whitehead K. (2020). Curaçao diagnostic criteria for hereditary hemorrhagic telangiectasia is highly predictive of a pathogenic variant in ENG or ACVRL1 (HHT1 and HHT2). Genet. Med..

[8] Gallione C.J., Richards J.A., Letteboer T., Rushlow D., Prigoda N.L., Leedom T.P., Ganguly A., Castells A., Van Amstel J.P., Westermann C. (2006). SMAD4 mutations found in unselected HHT patients. J. Med. Genet..

[9] Gallione C., Aylsworth A.S., Beis J., Berk T., Bernhardt B., Clark R.D., Clericuzio C., Danesino C., Drautz J., Fahl J. (2010). Overlapping spectra of SMAD4 mutations in juvenile polyposis (JP) and JP–HHT syndrome. Am. J. Med. Genet. A.

[10] Bayrak-Toydemir P., McDonald J., Markewitz B., Lewin S., Miller F., Chou L.S., Gedge F., Tang W., Coon H., Mao R. (2006). Genotype–phenotype correlation in hereditary hemorrhagic telangiectasia: Mutations and manifestations. Am. J. Med. Genet. A.

[11] Guttmacher A.E., Marchuk D.A., White R.I. (1995). Hereditary hemorrhagic telangiectasia. N. Engl. J. Med..

[12] Kasthuri R.S., Montifar M., Nelson J., Kim H., Lawton M.T., Faughnan M.E., Brain Vascular Malformation Consortium HHT Investigator Group (2017). Prevalence and predictors of anemia in hereditary hemorrhagic telangiectasia. Am. J. Hematol..

[13] Letteboer T.G., Mager J., Snijder R.J., Koeleman B.P., Lindhout D., Van Amstel J.P., Westermann C. (2006). Genotype-phenotype relationship in hereditary haemorrhagic telangiectasia. J. Med. Genet..

[14] Sabba C., Pasculli G., Lenato G., Suppressa P., Lastella P., Memeo M., Dicuonzo F., Guanti G. (2007). Hereditary hemorrhagic telangiectasia: Clinical features in ENG and ALK1 mutation carriers. J. Thromb. Haemost..

[15] Berg J., Porteous M., Reinhardt D., Gallione C., Holloway S., Umasunthar T., Lux A., McKinnon W., Marchuk D., Guttmacher A. (2003). Hereditary haemorrhagic telangiectasia: A questionnaire based study to delineate the different phenotypes caused by endoglin and ALK1 mutations. J. Med. Genet..

[16] Nishida T., Faughnan M.E., Krings T., Chakinala M., Gossage J.R., Young W.L., Kim H., Pourmohamad T., Henderson K.J., Schrum S.D. (2012). Brain arteriovenous malformations associated with hereditary hemorrhagic telangiectasia: Genotype-phenotype correlations. Am. J. Med. Genet. A.

[17] Pawlikowska L., Nelson J., Guo D.E., McCulloch C.E., Lawton M.T., Kim H., Faughnan M.E., Chakinala M., Clancy M., Brain Vascular Malformation Consortium HHT Investigator Group (2018). Association of common candidate variants with vascular malformations and intracranial hemorrhage in hereditary hemorrhagic telangiectasia. Mol. Genet. Genom. Med..

[18] Pawlikowska L., Nelson J., Guo D.E., McCulloch C.E., Lawton M.T., Young W.L., Kim H., Faughnan M.E. (2015). The ACVRL1 c.314-35A>G polymorphism is associated with organ vascular malformations in hereditary hemorrhagic telangiectasia patients with ENG mutations, but not in patients with ACVRL1 mutations. Am. J. Med. Genet. A.

[19] Kjeldsen A.D., Møller T., Brusgaard K., Vase P., Andersen P.E. (2005). Clinical symptoms according to genotype amongst patients with hereditary haemorrhagic telangiectasia. J. Intern. Med..

[20] Lesca G., Olivieri C., Burnichon N., Pagella F., Carette M.-F., Gilbert-Dussardier B., Goizet C., Roume J., Rabilloud M., Saurin J.-C. (2007). Genotype-phenotype correlations in hereditary hemorrhagic telangiectasia: Data from the French-Italian HHT network. Genet. Med..

[21] Sánchez-Martínez R., Iriarte A., Mora-Luján J.M., Patier J.L., López-Wolf D., Ojeda A., Torralba M.A., Juyol M.C., Gil R., Añón S. (2020). Current HHT genetic overview in Spain and its phenotypic correlation: Data from RiHHTa registry. J. Rare Disord..

[22] Krings T., Kim H., Power S., Nelson J., Faughnan M., Young W.L., Brain Vascular Malformation Consortium HHT Investigator Group (2015). Neurovascular manifestations in hereditary hemorrhagic telangiectasia: Imaging features and genotype-phenotype correlations. AJNR Am. J. Neuroradiol..

[23] Giordano P., Lenato G.M., Suppressa P., Lastella P., Dicuonzo F., Chiumarulo L., Sangerardi M., Piccarreta P., Valerio R., Scardapane A. (2013). Hereditary hemorrhagic telangiectasia: Arteriovenous malformations in children. Pediatrics.

[24] Al-Saleh S., Mei-Zahav M., Faughnan M., MacLusky I., Carpenter S., Letarte M., Ratjen F. (2009). Screening for pulmonary and cerebral arteriovenous malformations in children with hereditary haemorrhagic telangiectasia. Eur. Respir. J..

[25] Latino G.A., Al-Saleh S., Alharbi N., Edwards C., Faughnan M.E., Ratjen F. (2013). Prevalence of pulmonary arteriovenous malformations in children versus adults with hereditary hemorrhagic telangiectasia. Pediatrics.

[26] Krings T., Ozanne A., Chng S., Alvarez H., Rodesch G., Lasjaunias P. (2005). Neurovascular phenotypes in hereditary haemorrhagic telangiectasia patients according to age. Neuroradiology.

[27] Pasculli G., Resta F., Guastamacchia E., Suppressa P., Sabbà C. (2004). Health-related quality of life in a rare disease: Hereditary hemorrhagic telangiectasia (HHT) or Rendu–Osler–Weber Disease. Qual. Life Res..

[28] Plauchu H., De Chadarévian J.P., Bideau A., Robert J.M. (1989). Age—Related clinical profile of hereditary hemorrhagic telangiectasia in an epidemiologically recruited population. Am. J. Med. Genet..

[29] Pierucci P., Lenato G.M., Suppressa P., Lastella P., Triggiani V., Valerio R., Comelli M., Salvante D., Stella A., Resta N. (2012). A long diagnostic delay in patients with Hereditary Haemorrhagic Telangiectasia: A questionnaire-based retrospective study. J. Rare Disord..

[30] Pahl K.S., Choudhury A., Wusik K., Hammill A., White A., Henderson K., Pollak J., Kasthuri R.S. (2018). Applicability of the Curaçao criteria for the diagnosis of hereditary hemorrhagic telangiectasia in the pediatric population. Pediatrics.

[31] Akers A.L., Ball K.L., Clancy M., Comi A.M., Faughnan M.E., Gopal-Srivastava R., Jacobs T.P., Kim H., Krischer J., Marchuk D.A. (2013). Brain Vascular Malformation Consortium: Overview, progress and future directions. J. Rare Disord..

[32] Pahl K., Kasthuri R.S., Kitchens C.S., Kessler C.M., Konkle B.A., Streiff M.B., Garcia D.A. (2019). 11—Hereditary Hemorrhagic Telangiectasia. Consultative Hemostasis and Thrombosis.

[33] Morgan M., Zurin A., Harrington T., Little N. (2000). Changing role for preoperative embolisation in the management of arteriovenous malformations of the brain. J. Clin. Neurosci..

[34] Fulbright R.K., Chaloupka J.C., Putman C.M., Sze G.K., Merriam M.M., Lee G.K., Fayad P.B., Awad I.A., White R.I. (1998). MR of hereditary hemorrhagic telangiectasia: Prevalence and spectrum of cerebrovascular malformations. AJNR Am. J. Neuroradiol..

[35] Maher C.O., Piepgras D.G., Brown R.D., Friedman J.A., Pollock B.E. (2001). Cerebrovascular manifestations in 321 cases of hereditary hemorrhagic telangiectasia. Stroke.

[36] Vase P., Grove O. (1986). Gastrointestinal lesions in hereditary hemorrhagic telangiectasia. Gastroenterology.

[37] Jelsig A.M., Tørring P.M., Kjeldsen A., Qvist N., Bojesen A., Jensen U., Andersen M.K., Gerdes A.-M., Brusgaard K., Ousager L.B. (2016). JP–HHT phenotype in Danish patients with SMAD4 mutations. Clin. Genet..

[38] Mora-Luján J.M., Iriarte A., Alba E., Sánchez-Corral M.Á., Berrozpe A., Cerdà P., Cruellas F., Ribas J., Castellote J., Riera-Mestre A. (2020). Gastrointestinal Bleeding in Patients with Hereditary Hemorrhagic Telangiectasia: Risk Factors and Endoscopic Findings. J. Clin. Med..

[39] Letteboer T., Zewald R., Kamping E., De Haas G., Mager J., Snijder R., Lindhout D., Hennekam F., Westermann C., van Amstel J.P. (2005). Hereditary hemorrhagic telangiectasia: ENG and ALK-1 mutations in Dutch patients. Hum. Genet..

[40] Lesca G., Burnichon N., Raux G., Tosi M., Pinson S., Marion M.J., Babin E., Gilbert--Dussardier B., Rivière S., Goizet C. (2006). Distribution of ENG and ACVRL1 (ALK1) mutations in French HHT patients. Hum. Mutat..

[41] Gonzalez C., Mcdonald J., Stevenson D., Whitehead K., Petersen M., Presson A., Ding Q., Wilson K. (2017). Epistaxis in Children and Adolescents with Hereditary Hemorrhagic Telangiectasia. Laryngoscope.

